# Rational modulation of the innate immune system for neuroprotection in ischemic stroke

**DOI:** 10.3389/fnins.2015.00147

**Published:** 2015-04-29

**Authors:** Diana Amantea, Giuseppe Micieli, Cristina Tassorelli, María I. Cuartero, Iván Ballesteros, Michelangelo Certo, María A. Moro, Ignacio Lizasoain, Giacinto Bagetta

**Affiliations:** ^1^Section of Preclinical and Translational Pharmacology, Department of Pharmacy, Health and Nutritional Sciences, University of CalabriaRende, Italy; ^2^C. Mondino National Neurological InstitutePavia, Italy; ^3^Department of Brain and Behavioral Sciences, University of PaviaPavia, Italy; ^4^Unidad de Investigación Neurovascular, Departamento de Farmacología, Facultad de Medicina, Universidad Complutense de Madrid and Instituto de Investigación Hospital 12 de OctubreMadrid, Spain; ^5^Section of Neuropharmacology of Normal and Pathological Neuronal Plasticity, University Consortium for Adaptive Disorders and Head Pain, University of CalabriaRende, Italy

**Keywords:** cytokines, immune system, ischemic stroke, ischemic tolerance, macrophages, neutrophils, preconditioning

## Abstract

The innate immune system plays a dualistic role in the evolution of ischemic brain damage and has also been implicated in ischemic tolerance produced by different conditioning stimuli. Early after ischemia, perivascular astrocytes release cytokines and activate metalloproteases (MMPs) that contribute to blood–brain barrier (BBB) disruption and vasogenic oedema; whereas at later stages, they provide extracellular glutamate uptake, BBB regeneration and neurotrophic factors release. Similarly, early activation of microglia contributes to ischemic brain injury via the production of inflammatory cytokines, including tumor necrosis factor (TNF) and interleukin (IL)-1, reactive oxygen and nitrogen species and proteases. Nevertheless, microglia also contributes to the resolution of inflammation, by releasing IL-10 and tumor growth factor (TGF)-β, and to the late reparative processes by phagocytic activity and growth factors production. Indeed, after ischemia, microglia/macrophages differentiate toward several phenotypes: the M1 pro-inflammatory phenotype is classically activated via toll-like receptors or interferon-γ, whereas M2 phenotypes are alternatively activated by regulatory mediators, such as ILs 4, 10, 13, or TGF-β. Thus, immune cells exert a dualistic role on the evolution of ischemic brain damage, since the classic phenotypes promote injury, whereas alternatively activated M2 macrophages or N2 neutrophils prompt tissue remodeling and repair. Moreover, a subdued activation of the immune system has been involved in ischemic tolerance, since different preconditioning stimuli act via modulation of inflammatory mediators, including toll-like receptors and cytokine signaling pathways. This further underscores that the immuno-modulatory approach for the treatment of ischemic stroke should be aimed at blocking the detrimental effects, while promoting the beneficial responses of the immune reaction.

## Introduction

As highlighted by recent expression profiling studies, the majority of the genes acutely modulated in the blood of stroke patients are implicated in the regulation of the innate immune system (Tang et al., 2006; Barr et al., 2010; Oh et al., 2012; Brooks et al., 2014). Moreover, serum levels of markers of acute inflammation correlate with the severity of brain damage and neurological deficit (Fassbender et al., 1994; Smith et al., 2004; Basic Kes et al., 2008; Whiteley et al., 2009; Chang et al., 2010).

Indeed, the innate immune system plays a pivotal role in the evolution of ischemic cerebral injury, as soluble mediators (i.e., cytokines and chemokines) and specialized cells, activated in the brain or recruited from the periphery, actively participate to the detrimental processes implicated in tissue damage, as well as to the repair and regeneration phases (Kamel and Iadecola, 2012; Amantea et al., 2014a). The dualistic role exerted by several mediators of the immune reaction may explain why most anti-inflammatory approaches, conceived disregarding the potential beneficial function of the target, have failed to reach the clinical setting.

In addition, a subdued activation of the immune system has been involved in ischemic tolerance, since different preconditioning stimuli act by reprogramming the immune response, through the modulation of inflammatory mediators, including toll-like receptors (TLRs) and cytokine signaling pathways (Garcia-Bonilla et al., 2014a). This further underscores that promoting the endogenous neuroprotective reactions of the innate immune system represents an attractive opportunity to develop novel effective stroke therapeutics.

This review has been conceived to describe the role played by the diverse mediators of the innate immune system in ischemic brain damage, also highlighting their beneficial role with the ambition to stimulate more extensive research aimed at selectively targeting these processes.

## Cellular mediators of the immune response

### Resident immune cells

**Figure 1:**
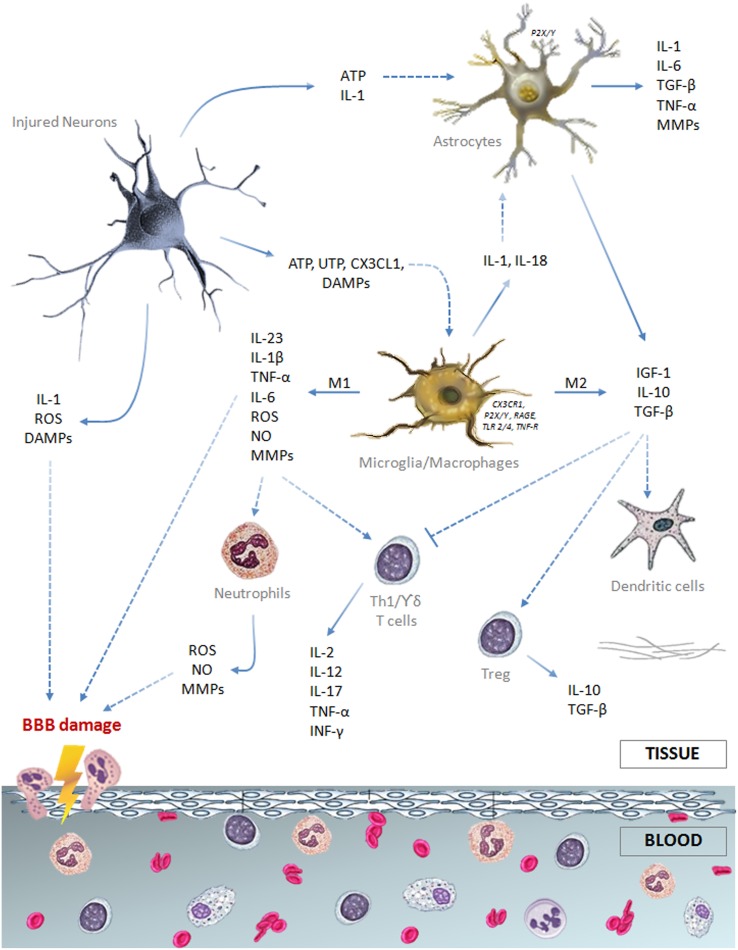
**Schematic drawing summarizing the major cellular and soluble mediators of the immune response elicited by an ischemic insult**.

All the cellular components of the neurovascular unit participate to the inflammatory reaction involved in ischemic stroke injury (Figure 1). On the intravascular side, platelets and the complement system are rapidly activated after vessel occlusion, thus providing the first trigger for the inflammatory response (Atkinson et al., 2006; Nieswandt et al., 2011). Elevated endothelial expression of the adhesion molecules P-selectin and intercellular adhesion molecule (ICAM)-1 promotes polymorphonuclear leukocytes (PMN) recruitment that exacerbates microvessel obstruction, also due to the reduced bioavailability of nitric oxide (NO) (Granger et al., 1989; Mori et al., 1992; Wong and Crack, 2008). Recently, Sreeramkumar et al. (2014) have demonstrated that the interaction between PMN and platelets within the microvasculature of infarcted brains is inhibited by blocking P-selectin glycoprotein ligand-1 (PSGL-1), and this correlates with a significant decrease in the infarct volume after permanent occlusion of the middle cerebral artery. Moreover, the ischemia-induced release of pro-inflammatory cytokines [e.g., interleukin (IL)-1 and tumor necrosis factor (TNF)] further promotes adhesion molecules expression and, together with the activation of proteases [i.e., matrix metalloproteases (MMPs)], prompts blood-brain barrier (BBB) breakdown leading to leukocytes extravasation in the injured brain (Ishikawa et al., 2004; Amantea et al., 2007; Yemisci et al., 2009; Yilmaz and Granger, 2010).

Astrocytes are the most abundant glial cells of the human brain and form part of the BBB. Ultrastructural studies have shown that pericapillary astrocyte end-feet are the first cellular elements to swell during cerebral ischemia (Dodson et al., 1977). Cytokines and reactive oxygen species (ROS), released by neurons and glial cells few minutes after the ischemic insult, alter molecular expression patterns in astrocytes and induce cellular hypertrophy, proliferation and scar formation (Sofroniew, 2009). Moreover, stimulation of P2Y(1) receptors by adenosine 5′-triphosphate (ATP), released or leaked from injured cells, prompts production of pro-inflammatory cytokines and chemokines by astrocytes via activation of a phosphorylated-p65 subunit (RelA)-mediated NF-κB pathway (Kuboyama et al., 2011). IL-1β and MMPs produced by perivascular astrocytes participate to BBB disruption and vasogenic edema (Rosenberg et al., 1998; del Zoppo and Hallenbeck, 2000; Amantea et al., 2010). Nevertheless, astrocytes exposed to an ischemic insult may also participate to neuroprotective and reparative responses (Table 1) by extracellular glutamate uptake (Stanimirovic et al., 1997; Rossi et al., 2007), BBB rebuilding (Kinoshita et al., 1990; del Zoppo, 2009) and neurotrophic factors release (Shen et al., 2010; Barreto et al., 2011). Accordingly, impairment of astrocyte function amplifies ischemic neuronal death (Nakase et al., 2003; Ouyang et al., 2007) and ablation of their reactivity and proliferation delays neurovascular remodeling and disrupts scar formation, negatively affecting functional recovery after focal cerebral ischemia in rodents (Nawashiro et al., 2000; Li et al., 2008; Hayakawa et al., 2010). Alternatively, proliferation of astrocytes induced by environmental enrichment or by pharmacological induction of signal transducer and activator of transcription factor (STAT)-3 phosphorylation ameliorates histological and functional outcomes in stroke models (Keiner et al., 2008; Amantea et al., 2011). Thus, reactive astrogliosis may exert a dualistic role on the propagation of ischemic brain damage, depending on the polarization of astrocytes toward specific phenotypes (Zamanian et al., 2012; Rusnakova et al., 2013) and on their interaction with surrounding neurons and microglia (Bezzi et al., 2001; Kang et al., 2012).

**Table 1:** **Dualistic effects of innate immune cells activated after ischemic brain injury**.

**Cell type**	**Detrimental effects**	**Beneficial effects**
Astrocytes	Production of inflammatory mediators (e.g., TNF-α, IL-1 and MMPs). Edema formation, inhibition of axon regeneration and BBB disruption.	Extracellular glutamate uptake, synthesis and release of neurotrophic factors. Glial scar formation, BBB rebuilding and neurovascular remodeling.
Microglia/macrophages	M-1 phenotype: production of pro-inflammatory cytokines, including TNF and IL-1, reactive oxygen and nitrogen species and proteases, such as MMPs.	M-2 phenotype: resolution of inflammation (IL-10 and TGF-β release, production of arginase and phagocytic activity). Late reparative processes by producing growth factors (IGF-1, brain-derived neurotrophic factor and glial cell line-derived neurotrophic factor).
Neutrophils	Microvessel obstruction, ROS production and release of MMPs that contribute to BBB damage and exacerbate inflammation.	N2 phenotype: promote resolution of inflammation
Dendritic cells	Up-regulation of MHC-II and co-stimulatory molecules that prompt activation of lymphocytes.	

After an ischemic insult, microglia, the resident immune cells of the central nervous system, is rapidly activated by ATP released by damaged neurons and other glial cells, acting on P2X7 receptors to prompt production and release of pro-inflammatory mediators (Melani et al., 2006; Dénes et al., 2007). Stimulation of microglia also relies on TLR4 stimulation, fractalkine receptor (CX3CR1) modulation and/or reduced CD200 receptor stimulation evoked by ischemia-induced disturbance of neuron-microglia cross-talk (Lehnardt et al., 2003; Dénes et al., 2008; Dentesano et al., 2012). Moreover, the increased release of specific neurotransmitters, such as glutamate and γ-aminobutyric acid, may elicit an inflammatory or a neuroprotective phenotype in microglia by signaling through Nox (Pocock and Kettenmann, 2007; Mead et al., 2012).

Microglial activation is accompanied by a substantial morphological transformation characterized in the early stages by retraction of cellular processes and enlargement of cell bodies, to ultimately acquire an amoeboid macrophage-like phenotype (Davis et al., 1994; Zhang et al., 1997; Schilling et al., 2003; Jung and Schwartz, 2012). As reported both in ischemia animal models (Zhang et al., 1997; Stoll et al., 1998; Dénes et al., 2007) and in stroke patients (Gerhard et al., 2005; Price et al., 2006), the reactivity and proliferation of microglia reach a peak few days after the insult and may persist for several weeks.

Reactive microglia may enhance inflammation and ischemic tissue injury via increased production and release of cytokines, such as IL-1 and TNF (Barone et al., 1997; Rothwell et al., 1997; Lambertsen et al., 2005; Amantea et al., 2010), reactive oxygen and nitrogen species (Green et al., 2001) and proteases (del Zoppo et al., 2007). These detrimental effects are underscored by the evidence that pharmacological- or microRNA-induced suppression of microglial activity limits ischemic cerebral injury (Hailer, 2008; Fagan et al., 2011; Zhang et al., 2012). However, other studies have demonstrated beneficial effects exerted by microglia in experimental stroke settings (Kitamura et al., 2004; Imai et al., 2007; Lalancette-Hébert et al., 2007). Indeed, by producing IL-10, transforming growth factor (TGF)-β and insulin-like growth factor (IGF)-1, microglia promotes resolution of the inflammatory reaction and reparative mechanisms involved in late tissue recovery (O'Donnell et al., 2002; Lalancette-Hébert et al., 2007; Neumann et al., 2008; Ransohoff and Cardona, 2010).

The reason for these apparently discrepant results can be found in the aptitude of microglia/macrophages to differentiate toward diverse phenotypes, depending on the dynamic evolution of the ischemic damage (Clausen et al., 2008; Perego et al., 2011). Indeed, the classic pro-inflammatory M1 phenotype is activated by interferon (INF)-γ or through TLRs modulation, whereas the alternatively activated M2 phenotype is induced by regulatory factors, including interleukins 4, 10, 13, or TGF-β (Italiani and Boraschi, 2014) (Table 1). Early after an ischemic insult, local microglia assumes the M2 “beneficial” phenotype, to then develop into a pro-inflammatory M1 phenotype prompted by the ischemic neurons (Hu et al., 2012). Thus, in addition to the classical approach aimed at suppressing detrimental M1 functions (i.e., production of TNF-α, IL-1β, monocyte chemoattractant protein (MCP)-1, macrophage inflammatory protein (MIP)-1α, and IL-6), preservation of the alternatively activated M2 phenotype may represent an innovative strategy for stroke neuroprotection, as demonstrated in mice lacking the class-A scavenger receptor (Xu et al., 2012) or the myeloid-specific mineralcorticoid receptor (Frieler et al., 2011). Given the similarities between local microglia and macrophages, as well as the ability of microglia to develop active phenotypes indistinguishable from circulating macrophages, further insights into their polarization will be given in the following section.

### Blood-borne cells

Genomic profile studies indicate a critical role of innate immunity in regulating stroke response and recovery. The genes identified in these profiles are involved in immune signaling at different levels, including the cerebral microenvironment, the vasculature and, most notably, the peripheral circulation (Brooks et al., 2014). In the first analysis performed in stroke patients, Moore et al. (2005) detected the expression of genes associated with the regulation of the cerebral microenvironment in peripheral blood mononuclear cells (PMBCs), thus supporting the important finding that peripheral blood is reflective of changes in the brain.

Interestingly, the majority of the genes identified in all the stroke-specific profiles published to date are related to the immune system (Moore et al., 2005; Tang et al., 2006; Barr et al., 2010; Oh et al., 2012). Among these studies, Brooks et al. (2014) recently identified a panel of overlapping genes, significantly expressed within 3 h from stroke onset, including arginase1 (ARG1), carbonic anhydrase 4 (CA4), lymphocyte antigen 96 (LY96), matrix metalloproteinase 9 (MMP9), and S100 calcium binding protein A12 (S100A12). Specifically, four of these genes (ARG1, LY96, MMP9, S100A12) are implicated in the innate immune response, the fifth (CA4) being highly expressed in the BBB (Brooks et al., 2014).

These findings strongly support the theory that the immune response to the ischemic insult engages specialized circulating cells, such as neutrophils, macrophages, dendritic cells and T lymphocytes, that are recruited and migrate to the brain, upon activation triggered by soluble factors released by injured and dying cells (Price et al., 2004; Buck et al., 2008; Felger et al., 2010; Yilmaz and Granger, 2010; Kamel and Iadecola, 2012). Infiltrating leukocytes affect the evolution of tissue damage by releasing a series of mediators including purines, ROS and danger associated molecular patterns (DAMPs), such as high mobility group box (HMGB)-1 protein, heat shock protein 60, β-amyloid, DNA or RNA immune complexes (Amantea et al., 2014a).

Interestingly, the majority of the genes acutely regulated in the blood of stroke patients are expressed in neutrophils and, to a lesser extent, in macrophages (Tang et al., 2006). Accordingly, these are the first cells to infiltrate the ischemic brain, reaching a peak within 24–72 h after the insult (Clark et al., 1994; Garcia et al., 1994; Gelderblom et al., 2009). In patients, higher peripheral leukocyte and neutrophil counts, but not lymphocyte counts, are associated with larger infarct volumes (Buck et al., 2008), and brain accumulation of neutrophils correlates with poor neurological outcome and brain damage severity both in humans (Akopov et al., 1996) and in rodents (Matsuo et al., 1994a,b; Connolly et al., 1996; Atochin et al., 2000). In fact, neutrophils prompt microvessel obstruction/thrombosis (del Zoppo et al., 1991; Ritter et al., 2005), production of ROS and release of MMPs (Justicia et al., 2003; Gidday et al., 2005; Bao Dang et al., 2013); thus, their modulation may represent a useful strategy to ameliorate stroke outcome. This may be achieved by pharmacological approaches, as well as by remote ischemic preconditioning, as documented by the evidence that forearm transient ischemia reduces neutrophil function, including adhesion, exocytosis, phagocytosis and cytokine secretion (Shimizu et al., 2010).

Interestingly, recent findings have highlighted the ability of neutrophils to polarize toward beneficial N2 phenotypes. In the setting of stroke, neutrophil reprogramming can be induced by activation of the nuclear peroxisome proliferator-activated receptor (PPAR)-γ (Cuartero et al., 2013). Thus, despite promoting early neutrophil infiltration to the ischemic core, the PPAR-γ agonist rosiglitazone provides neuroprotection and resolution of inflammation after experimental stroke induced by permanent MCAo by promoting N2-polarization and increased neutrophil clearance (Cuartero et al., 2013).

Likewise neutrophils, hematogenous macrophages infiltrating the ischemic brain (Schilling et al., 2003; Jander et al., 2007), exert a dualistic role on the evolution of tissue damage (Frieler et al., 2011; Hu et al., 2012; Xu et al., 2012). In fact, they possess the ability to switch between the classically activated M1 phenotype and alternatively activated M2 phenotypes (Ballesteros et al., 2014a) (Table 1). The M1 cells initiate and sustain inflammation by releasing neurotoxic factors and ROS that underlie macrophage/microglia-mediated neurotoxicity after stroke; whereas, M2-polarized cells are involved in beneficial responses by clearing debris and by promoting angiogenesis, tissue remodeling and repair (Gliem et al., 2012; Shechter and Schwartz, 2013). Up-regulation of M2 markers observed in the ischemic brain (Frieler et al., 2011; Perego et al., 2011; Hu et al., 2012; Zarruk et al., 2012; Ballesteros et al., 2014a) is due to an increased cerebral infiltration of alternatively activated blood-borne monocytes (Perego et al., 2011), as well as to the ability of local microglia/macrophages to assume an M2 phenotype (Hu et al., 2012). Indeed, local microglia and newly recruited macrophages assume the M2 phenotype at early stages of ischemic stroke but, upon priming by ischemic neurons, gradually transform into the M1 phenotype (Hu et al., 2012). The exact mechanisms that control macrophage polarization in the setting of stroke have not been fully elucidated, as well as it is not clear whether the acquisition of a specific phenotype involves recruitment of circulating precursors or *in situ* cell re-instruction. Endogenous production of the M2-polarizing cytokine IL-4, triggered by MCAo in mice, has been shown to promote Th2 polarization and, thus, beneficial effects on stroke outcome (Xiong et al., 2011). Further studies have shown that a subpopulation of bone marrow-derived monocytes/macrophages, recruited via CCR2 and acting through TGF-β1, maintains the integrity of the neurovascular unit in murine stroke models (Gliem et al., 2012).

To date, only few studies have assessed the therapeutic benefits of reducing the M1/M2 ratio in stroke setting. Frieler et al. (2011) showed that deficiency of the mineralcorticoid receptor (MR) decreases the expression of M1 markers, while preserving the ischemia-induced expression of M2 markers. The resulting elevation of M2 polarized myeloid cells in the ischemic brain was correlated with a better stroke outcome in MR^−/−^ mice (Frieler et al., 2011). Similarly, deficiency of the fractalkine receptor CX3CR1 has been associated with a protective inflammatory milieu, characterized by the promotion of M2 polarization markers (Fumagalli et al., 2013). PPAR-γ-mediated CD36 up-regulation has been involved in the modulation of microglia phenotype, promoting phagocytosis of apoptotic neutrophils, and thus contributing to the resolution of inflammation after stroke (Ballesteros et al., 2014b). By contrast, elevation of M1/M2 ratio promoted by the class A scavenger receptor expressed in microglia/macrophages has been associated with exacerbation of ischemic brain injury (Xu et al., 2012). At variance with the latter, the selective cannabionoid receptor 2 agonist, JWH-133, provides neuroprotection in the acute phase of ischemic stroke by reducing microglia activation, without affecting M2 polarization (Zarruk et al., 2012).

Although preclinical findings strongly suggest the therapeutic usefulness of M2-polarizing agents, the exact mechanisms modulating M1/M2 ratio need further investigation and their relevance for stroke outcome in human stroke has to be validated.

At later stages after the ischemic insult, a significant elevation of dendritic cells occurs in the injured brain hemisphere, reaching a peak 72 h after the insult (Kostulas et al., 2002; Reichmann et al., 2002; Gelderblom et al., 2009). Both peripheral and brain resident dendritic cells are pivotally involved in bridging innate and adaptive immunity by up-regulating major histocompatibility complex (MHC)-II and co-stimulatory molecules that contribute to the activation of lymphocytes (Gelderblom et al., 2009; Felger et al., 2010). Moreover, resident dendritic cells participate in orchestrating the early local immune response and in the late recruitment of lymphocytes (Felger et al., 2010) following activation by INF-γ (Gottfried-Blackmore et al., 2009). Brain infiltration of T lymphocytes occurs relatively late (i.e., 3–7 days) after ischemia (Jander et al., 1995; Schwab et al., 2001; Gelderblom et al., 2009); nevertheless, these cells contribute to the progression of brain damage (Yilmaz et al., 2006; Hurn et al., 2007; Jin et al., 2010), exerting distinct effects depending on the specific cell subset recruited (Amantea et al., 2014a). Recent evidence demonstrates that the functional sphingosine-1-phosphate receptor agonist FTY720 (fingolimod), minimizes brain damage and functional deficits in experimental stroke (Shichita et al., 2009; Hasegawa et al., 2010; Wei et al., 2011). Interestingly, the putative elevation of the incidence of bacterial pneumonia caused by the inhibition of the adaptive immunity by fingolimod does not seem to be actually relevant for the neuroprotective effects of the drug (Pfeilschifter et al., 2011).

Thus, the ischemic insult is associated to a relevant activation of the innate immune system, involving both local and blood-borne specialized cells that, upon recruitment, modulate the cerebral inflammatory response to stroke and set the stage for the activation of adaptive immunity (Figure 1).

## Molecular mediators of the immune response

### Receptors

As stated above, activation of P2X7 receptors on microglia prompts the processing and release of pro-inflammatory cytokines (Brough et al., 2002; Melani et al., 2006). Moreover, overactivation of P2X7 receptors is involved in excitotoxic neuronal death (Arbeloa et al., 2012) and participates to ischemia-induced damage to olygodendrocytes and myelin (Domercq et al., 2010).

Activation of TLRs by HMGB1, peroxiredoxin (Prx) proteins and other DAMPS, plays an important role in ischemic brain injury (Fossati and Chiarugi, 2007; Shichita et al., 2012a,b; Pradillo et al., 2014). In particular, TLR2 and TLR4 crucially contribute to the induction of the inflammatory response and to the evolution of brain damage, as documented by the evidence that TLR2- or TLR4-deficiency is associated to reduced ischemic brain damage and to suppression of ischemia-induced expression and release of inflammatory cytokines (Tang et al., 2007; Hyakkoku et al., 2010). Indeed, TLR4 deficient mice display significant suppression of IκB phosphorylation, NFκB activity, pro-inflammatory mediators, including TNF-α and IL-6 (Cao et al., 2007; Hyakkoku et al., 2010) and the enzymes inducible nitric oxide synthase (NOS) and cyclooxygenase (COX)-2 (Caso et al., 2007, 2008).

The relevance of TLR2 and TLR4 has also been demonstrated in ischemic stroke patients, since up-regulation of these receptors is associated with greater inflammatory responses and with poor functional outcome (Brea et al., 2011). The stimulation of macrophages and T cells by TLRs-associated pathways induces strong inflammatory responses (Shichita et al., 2012a). Following cerebral ischemia, the activation of TLR4 by HMGB-1 induces MMP-9 up-regulation in neurons and astrocytes (Qiu et al., 2010) and promotes detrimental effects by macrophages infiltrating the injured brain (Yang et al., 2011). In fact, cerebral microinjection of HMGB-1 increases the transcript levels of pro-inflammatory mediators and sensitizes the tissue to ischemic injury (Faraco et al., 2007). In addition, deficiency of TLR4 in young animals subjected to focal cerebral ischemia, promotes subventricular zone cell proliferation, increasing the number of the transit-amplifying cells (type C cells; prominin-1+/EGFR+/nestin- cells) at 24 and 48 h, of proliferating immature (BrdU+) cells at 7d and of neuroblast cells (type A cells; doublecortin+ cells) at 14d (Moraga et al., 2014). Despite a negative effect on SVZ cell proliferation, TLR4 plays an important role in stroke-induced neurogenesis by promoting neuroblasts migration and increasing the number of new cortical neurons after stroke (Moraga et al., 2014).

Although the exact mechanisms by which TLRs modulate the evolution of ischemic brain injury have not been fully elucidated, pharmacological inhibition of TLR2 and TLR4 and/or blockade of some of their endogenous ligands (i.e., cellular fibronectin or heat shock protein 60), represent promising therapeutic options, effective in reducing the inflammatory response to stroke injury (Brea et al., 2011).

Paradoxically, by reprogramming TLRs signaling, stimulation of TLRs before ischemia leads to suppression of pro-inflammatory responses and to enhanced expression of numerous anti-inflammatory mediators that collectively contribute to neuroprotection (Pradillo et al., 2009; Vartanian et al., 2011). Activation of TLR4 by low doses of LPS reduces synthesis and release of some pro-inflammatory cytokines, and inhibits microglial activation and neutrophil infiltration, thus reducing ischemic brain injury (Rosenzweig et al., 2004; Pradillo et al., 2009). Tolerance to brain ischemia induced by low doses of the major TLR4 ligand, LPS, administered 1–3 days before the insult, has been demonstrated in several experimental stroke models (Tasaki et al., 1997; Hickey et al., 2007; Yu et al., 2010). Recent work has also demonstrated that ischemic preconditioning reduces brain damage from permanent middle cerebral artery occlusion in mice by increasing expression of TLR3 in cortical astrocytes (Pan et al., 2014). Therefore, induction of ischemic tolerance by subdued TLRs activation represents an interesting opportunity to exploit these receptors for stroke therapy.

The receptor for advanced glycation end products (RAGE) is a member of the immunoglobulin family of cell surface receptors and has been implicated in the development and progression of stroke. The full-length, membrane-bound RAGE isoform (fl-RAGE) is mainly expressed in neurons and in microglia/macrophages. Up-regulation of this receptor has been documented after both permanent and transient focal cerebral ischemia in rodents (Qiu et al., 2008; Zhai et al., 2008; Hassid et al., 2009) and in the ischemic hemisphere of stroke patients (Hassid et al., 2009). More specifically, ischemia-induced modifications of the expression of RAGE and its isoforms in the brain strongly depends on the intensity and on the propagation of the insult; showing a distinct modulation in the core and penumbra regions (Greco et al., 2012, 2014). By activating fl-RAGE on microglia/macrophages, HMGB-1 protein released from dying neurons, contributes to the development of ischemic brain damage (Muhammad et al., 2008; Qiu et al., 2008). Conversely, blockade of fl-RAGE signaling promotes cell survival and reduces stroke infarct volume in animal models (Kim et al., 2006; Liu et al., 2007; Muhammad et al., 2008).

A reduced plasma level of soluble RAGE isoforms (sRAGE) has been reported in rats subjected to either transient or permanent focal brain ischemia (Greco et al., 2012, 2014). This is of significance since sRAGE, generated either by alternative splicing or by proteolysis of the full-length form, effectively bind AGEs, thereby competing with the cell surface fl-RAGE, thus providing a “decoy” function that may counteract the detrimental effects of receptor signaling in neurons (Koyama et al., 2007; Muhammad et al., 2008; Tang et al., 2013). Interestingly, the reduction of sRAGE levels induced by transient MCAo in rats, is minimized by pre-treatment with a neuroprotective dose of the poly(ADP-ribose) polymerase (PARP) inhibitor PJ34 (Greco et al., 2014), suggesting that sRAGE may represent a useful biomarker of stroke severity and of effective neuroprotective treatment. In patients, some studies have reported an association between circulating sRAGE levels and brain infarct volume, stroke severity (Park et al., 2004; Yokota et al., 2009) and inflammatory status (Cui et al., 2013), others have suggested that sRAGE levels at onset may predict cognitive impairment after cerebral ischemia (Qian et al., 2012).

### Inflammatory cytokines

The inflammatory response to ischemic brain injury is orchestrated by a variety of cytokines released by resident brain cells, such as neurons and glia, and by blood-borne immune cells. TNF, IL-1 and IL-6 strongly affect the development of ischemic brain damage in animal models, and their levels are increased in the blood and in the cerebrospinal fluid of stroke patients (Lambertsen et al., 2012), thus attracting considerable interest as putative markers of stroke severity and neurologic outcome (Emsley et al., 2007; Jickling and Sharp, 2011). By contrast, other cytokines, including IL-10 and TGF-β exert immunoregulatory and anti-inflammatory effects, thus promoting reparative processes. In addition, mild systemic inflammation induced by remote preconditioning is neuroprotective in stroke models (Petcu et al., 2008) and several soluble mediators of the innate immune system have been involved in ischemic tolerance produced by diverse preconditioning stimuli (Garcia-Bonilla et al., 2014a).

A significant and rapid up-regulation of TNF-α occurs following focal cerebral ischemia both in animal models and in stroke patients. In fact, expression of this cytokine is elevated in neurons during the first hours after the insult; whereas, at later stages, it is increased in microglia/macrophages and in blood-borne immune cells (Gregersen et al., 2000; Dziewulska and Mossakowski, 2003). Accordingly, recent flow cytometry experiments have demonstrated that the major source of this cytokine in the stroke-lesioned mouse brain is microglia and macrophages (Clausen et al., 2008; Lambertsen et al., 2012).

TNF-α is believed to play a detrimental role in ischemic injury, since its neutralization with specific monoclonal antibodies or binding proteins provides neuroprotection in experimental stroke models (Barone et al., 1997; Nawashiro et al., 1997; Lavine et al., 1998; Lambertsen et al., 2012). Nevertheless, studies from transgenic animals demonstrate that the cytokine may also exert beneficial effects through the activation of p55 TNF receptor (TNF-RI) (Bruce et al., 1996; Gary et al., 1998; Taoufik et al., 2007; Lambertsen et al., 2009). In rodents, expression of TNF-RI is elevated in neurons and in non-neuronal cells few hours after MCAo, whereas up-regulation of TNF-RII occurs from 24 h after injury in resident microglia and infiltrating macrophages (Botchkina et al., 1997; Dziewulska and Mossakowski, 2003; Yin et al., 2004; Pradillo et al., 2005; Lambertsen et al., 2007). Despite its predominant inflammatory role, TNF-RII has also been implicated in neuroprotection (Marchetti et al., 2004), and this further complicates the interpretation of the pleiotropic effects of TNF in ischemic neuronal damage (Hallenbeck, 2002; McCoy and Tansey, 2008).

Moreover, the TNF pathway has been involved in ischemic, hypoxic, endotoxic and exercise-induced preconditioning (Garcia-Bonilla et al., 2014a). In fact, LPS-preconditioned TNF-α null mice are not protected from ischemic brain injury (Rosenzweig et al., 2007) and administration of a neutralizing TNF binding protein nullifies the beneficial effect of LPS pre-treatment in spontaneously hypertensive rats subjected to permanent MCAo (Tasaki et al., 1997). Paradoxically, TNF-α has a dualistic effect in stroke, since it's up-regulation has been shown to underlie LPS-induced tolerance in mice subjected to focal cerebral ischemia, whereas suppression of TNF-α signaling during ischemia confers neuroprotection after LPS preconditioning (Rosenzweig et al., 2007). Up-regulation of TNF-α converting enzyme (TACE) and increased serum levels of TNF-α have also been involved in preconditioning induced by prolonged and intermittent normobaric hyperoxia in rats subjected to MCAo (Bigdeli and Khoshbaten, 2008; Bigdeli et al., 2008). In rats, the increased expression of TNF-α lasting for 2–3 weeks of physical activity underlies amelioration of downstream inflammatory events, reduced BBB disruption and the consequent ischemic neuroprotection induced by exercise preconditioning (Ding et al., 2005, 2006; Guo et al., 2008b).

IL-1 is a proinflammatory cytokine that plays a pivotal role in the neurodegenerative processes triggered by the ischemic insult (Olsson et al., 2012; Dénes et al., 2013). Cerebral levels of both IL-1α and IL-1β are elevated within hours of reperfusion after focal ischemia (Hara et al., 1997; Amantea et al., 2007, 2010; Luheshi et al., 2011). IL-1α is mainly induced in microglia, whereas IL-1β can be released by all the elements of the neurovascular unit, including neurons, astrocytes, microglia/macrophages and endothelial cells, being its cellular source strongly dependent on the spatio-temporal evolution of the damage (Amantea et al., 2010; Luheshi et al., 2011; Giles et al., 2015). In astrocytes and microglia, ischemia-induced production of IL-1β involves activation of TLR4 (Simi et al., 2007) and p38 mitogen-activated protein kinase (MAPK) (Walton et al., 1998; Irving et al., 2000). Interestingly, under ischemic conditions, caspase-1-independent pathways seem to contribute to IL-1β maturation. Accordingly, it has been recently demonstrated that early elevation of IL-1β in the ischemic cortex of rats subjected to transient MCAo is not associated with caspase-1 activation, whereas production of the mature cytokine is strongly dependent on gelatinases, i.e., MMP2 and MMP-9 activity (Amantea et al., 2007, 2014b).

It is intriguing to observe that IL-1 is not directly toxic to healthy neurons, whereas it may become harmful via the modulation of other elements of the neurovascular unit, such as astrocytes and the endothelium. IL-1 stimulates astrogliosis (Herx and Yong, 2001) and prompts the release of cytokines, chemokines (Andre et al., 2005) and the activation of MMP-9 in astrocytes (Thornton et al., 2008). Furthermore, this cytokine induces endothelial expression of adhesion molecules, including ICAM-1 and vascular cell adhesion molecule (VCAM)-1, that together with the local stimulation of the release of chemokines, promotes neutrophil adhesion and infiltration in the injured hemisphere (Thornton et al., 2011; Allen et al., 2012).

Despite their established detrimental roles, both IL-1α and IL-1β have been shown to underlie tolerance to global ischemia induced by common carotid artery occlusion in gerbil (Ohtsuki et al., 1996). Moreover, ischemic preconditioning produced by bilateral common carotid artery occlusion protects mice against subsequent MCAo injury by differentially regulating cortical IL-1β and IL-1 receptor antagonist (IL-1ra) expression in order to promote a shift toward an anti-inflammatory state that contributes to neuroprotection (Shin et al., 2009). The regulation of the expression and of the effects of IL-1 has also been implicated in LPS preconditioning via the TLR4 signaling pathway (Gong et al., 2014).

Other cytokines involved in the evolution of brain infarction and contributing to aggravate neurological functions are IL-17 and IL-23 (Shichita et al., 2009; Konoeda et al., 2010; Ma et al., 2013; Swardfager et al., 2013). IL-17A secreted by γδ T cells promotes neutrophil recruitment and its blockade with specific antibodies exerts neuroprotection (Gelderblom et al., 2012). Despite the presence of IL-17A-positive lymphocytes in autoptic brain of stroke patients, IL-17 blood level does not seem to be a good predictor of stroke outcome as compared to other cytokines such as IL-6 (Zeng et al., 2013). In fact, plasma and cerebrospinal fluid levels of IL-6 correlate with stroke severity and poor clinical outcome in patients (Smith et al., 2004; Waje-Andreassen et al., 2005; Whiteley et al., 2009) and reduced blood concentrations of this cytokine have been correlated to the improved outcome induced by treatment with IL-1ra (Emsley et al., 2005). By contrast, in stroke animal models, IL-6 appears to play a neuroprotective role (Matsuda et al., 1996; Loddick et al., 1998; Herrmann et al., 2003), suggesting that its rapid and persistent elevation in the ischemic brain (Suzuki et al., 1999; Ali et al., 2000) may represent a compensatory mechanism to counteract the damaging effects of the insult. In fact, by activating its receptor and the downstream phosphorylation of STAT-3, IL-6 may induce neuroprotection (Yamashita et al., 2005; Jung et al., 2011). Moreover, IL-6 enhances the effectiveness of cell transplantation therapy in ischemic stroke, by reprogramming neural stem cells to tolerate oxidative stress and to induce angiogenesis through STAT-3 activation (Perini et al., 2001).

### Anti-inflammatory cytokines

While plasma concentrations of detrimental cytokines are elevated, levels of IL-10, associated with better outcome, are decreased in patients (Perini et al., 2001; Vila et al., 2003; Basic Kes et al., 2008). Results from animal models demonstrate that IL-10 represents a major downregulator of the detrimental effects of proinflammatory mediators during stroke and modulates neuronal vulnerability to excitotoxic ischemic damage (Spera et al., 1998; Grilli et al., 2000; Frenkel et al., 2005).

Another cytokine playing beneficial effects in cerebral ischemia is TGF-β (Gliem et al., 2012). Levels of this cytokine are elevated in the blood of patients 1 day after ischemic stroke (Yan et al., 2012) and in activated astrocytes and microglia/macrophages of the ischemic brain for at least 1 week in animal models (Lehrmann et al., 1998; Yamashita et al., 1999; Doyle et al., 2010). In rats, administration of a TGF-β antagonist aggravates brain damage caused by focal cerebral ischemia (Ruocco et al., 1999); whereas, in mice, intranasal delivery of this cytokine after stroke reduces infarct volume and increases neurogenesis in the subventricular zone (Ma et al., 2008). The beneficial effects of TGF-β involve both anti-inflammatory effects, including inhibition of brain elevation of MCP-1 and MIP-1α (Pang et al., 2001), but also induction of glial scar formation (Logan et al., 1994) and anti-apoptotic effects (Zhu et al., 2002).

In mice, preconditioning with low dose LPS is associated with upregulation of anti-inflammatory cytokines, including TGF-β in brain and IL-10 in blood (Vartanian et al., 2011).

### Chemokines

In patients with acute ischemic stroke, serum levels of stromal cell-derived factor (SDF)-1α have been correlated with favorable long-term outcome (Kim et al., 2012) and this chemokine has been suggested to be a predictor of future stroke (Schutt et al., 2012). SDF-1α promotes bone marrow-derived cell targeting to the ischemic brain and improves local cerebral blood flow (Cui et al., 2007; Shyu et al., 2008), thus representing a promising target to implement stem cell therapy in patients. In fact, in the injured hemisphere, it activates CXCR4 on neural progenitor cells guiding their specific migration to the site of damage (Robin et al., 2006). Moreover, SDF-1α participates to the regulation of post-ischemic inflammation and it is involved in neurovascular repair (Wang et al., 2012). Despite these documented beneficial effects, some detrimental roles have also been suggested to be mediated by this chemokine, since pharmacological blockade of CXCR4 is neuroprotective in stroke by reducing BBB damage and inflammatory processes (Huang et al., 2013). Soluble fractalkine (CX3CL1) released by neurons upon an ischemic insult controls leukocyte trafficking and participates to the activation and chemoattraction of microglia to the injured brain via the activation of CX3CR1 receptors (Tarozzo et al., 2002; Dénes et al., 2008; Zhu et al., 2009). The detrimental effects of this chemokine are mediated by IL-1β and TNF-α in stroke mouse models (Soriano et al., 2002; Dénes et al., 2008). By contrast, in ischemic stroke patients, higher plasma concentrations of fractalkine are associated with better outcome and with low levels of systemic markers of inflammation (Donohue et al., 2012). In fact, by inhibiting caspase-3 and by activating adenosine receptors, exogenous administration of fractalkine is neuroprotective in wild-type rodents undergone permanent ischemia (Cipriani et al., 2011; Rosito et al., 2014). The apparent discrepancies between this latter finding and those produced in transgenic animals may be explained by the altered microglia responsiveness to fractalkine in the absence of constitutive fractalkine-CX3CR1 signaling (Cipriani et al., 2011).

Among the chemokines implicated in cerebral ischemia, MCP-1 (also known as CCL2) and MIP-1α have been shown to play an important role in promoting tissue damage via recruitment of inflammatory cells (Wang et al., 1995; Che et al., 2001; Takami et al., 2001; Minami and Satoh, 2003). The mRNA levels of both chemokines are elevated in the ischemic brain of rodents and MCP-1 levels are also increased in the cerebrospinal fluid of stroke patients (Losy and Zaremba, 2001). Mice lacking MCP-1 or its receptor, CCR2, display reduced infarct volume along with impaired leukocyte recruitment and reduced expression of inflammatory mediators in the injured brain (Hughes et al., 2002; Dimitrijevic et al., 2007; Strecker et al., 2011; Schuette-Nuetgen et al., 2012). Conversely, overexpression of MCP-1 prompts exacerbation of brain injury and increased cerebral recruitment of inflammatory cells (Chen et al., 2003). Moreover, MCP-1, as well as SDF-1α, promotes migration of newly formed neuroblasts from neurogenic regions to ischemic damaged areas (Robin et al., 2006; Yan et al., 2007).

Up-regulation of MCP-1 has been shown to underlie hypoxic preconditioning-induced stroke tolerance in mice (Stowe et al., 2012; Wacker et al., 2012); whereas, activation of CCR2 is involved in the neuroprotective effects exerted by both ischemic preconditioning and post-conditioning in mice subjected to global cerebral ischemia (Rehni and Singh, 2012).

Monocyte chemotactic protein-induced protein 1 (MCPIP1) deficiency exacerbates ischemic brain damage by upregulation of proinflammatory cytokines and this Zn finger-containing immunoregulatory protein also participates in LPS- and electroacupuncture-induced ischemic stroke tolerance (Liang et al., 2001; Jin et al., 2013).

### Enzymes: MMPs, COX, and NOS

MMPs play a crucial role in the evolution of the inflammatory response to ischemic brain injury (Cunningham et al., 2005). Stroke-induced damage to the BBB and hemorrhagic transformation are both induced by the activation of the gelatinases, MMP-2 and MMP-9, as demonstrated in animal models (Romanic et al., 1998; Rosenberg et al., 1998; Asahi et al., 2000) and in patients (Horstmann et al., 2003; Rosell et al., 2006, 2008). RNA-expression levels of MMP-9 in circulating monocytes have been correlated with the brain infarct lesion in stroke patients (Ulrich et al., 2013); while, serum levels of this enzyme have been associated with clinical diffusion mismatch (Rodríguez-Yáñez et al., 2011).

Cerebral expression and activity of gelatinases increase very early after an ischemic insult, with a specific cellular expression pattern dependent on the spatio-temporal evolution of the damage (Rosenberg et al., 1998; Planas et al., 2001; Yang et al., 2007; Amantea et al., 2008). Pharmacological inhibition of gelatinases, as well as gene deletion of MMP-9, reduces infarct volume caused by focal cerebral ischemia in rodents (Romanic et al., 1998; Asahi et al., 2000; Gasche et al., 2001; Amantea et al., 2007, 2014b). The mechanisms by which gelatinases contribute to ischemic brain damage include disruption of BBB integrity, hemorrhagic transformation and white matter myelin degradation (Cunningham et al., 2005). Moreover, MMPs and their endogenous inhibitors (TIMPs) regulate neuronal cell death through modulation of excitotoxicity (Jourquin et al., 2003), DNA repairing enzymes (Hill et al., 2012), anoikis (Gu et al., 2002), calpain activity (Copin et al., 2005) and production of neurotoxic products (Gu et al., 2002; Zhang et al., 2003), including pro-inflammatory cytokines (Amantea et al., 2007, 2014b). Moreover, the degradation of tight junction proteins claudin-5 and occludin by MMPs prompts hemorrhagic transformation, suggesting that these enzymes may represent a promising target for reducing the hemorrhagic complications associated with thrombolytic therapy (Yang and Rosenberg, 2011; Liu et al., 2012).

The up-regulation of the extracellular MMP inducer (EMMPRIN) occurring in peri-ischemic regions 2–7 days after focal ischemia in mice is coincident with the delayed increase of MMP-9, suggesting its involvement in neurovascular remodeling (Zhu et al., 2008). In fact, the late activation of MMP-9 promotes vascular endothelial growth factor (VEGF) signaling, contributing to neuronal survival and new vessels formation (Zhao et al., 2006). Thus, MMPs play a dual role in stroke injury, including early detrimental effects and beneficial roles at later stages after the insult. Moreover, MMPs participate to the protective response evoked by several preconditioning stimuli. In fact, tolerance produced by ischemic preconditioning or pre-ischemic exercise has been shown to be associated with downregulation of MMP-9 and subsequent amelioration of brain oedema and BBB disruption in rats undergone focal cerebral ischemia (Zhang et al., 2006; Davis et al., 2007; Guo et al., 2008a). Similarly, the neuroprotective action of TNF-α induced by pre-ischemic physical exercise has been demonstrated to occur via reduced MMP-9 activity and amelioration of BBB dysfunction in rats subjected to transient MCAo, through the involvement of extracellular signal-regulated kinase 1 and 2 (ERK1/2) phosphorylation (Guo et al., 2008b; Chaudhry et al., 2010). Reduced activity of gelatinases was also associated with the neuroprotective effects exerted by hyperbaric oxygen preconditioning in hyperglycemic rats subjected to MCAo (Soejima et al., 2013).

After an ischemic insult, the expression of COX-2 is elevated in neurons, vascular cells and neutrophils, as demonstrated both in stroke patients and in animal models (Nogawa et al., 1997; Iadecola et al., 1999, 2001; Chakraborti et al., 2010). This enzyme contributes to post-ischemic inflammation through the production of toxic prostanoids and superoxide, and its deficiency or pharmacological inhibition leads to reduce BBB damage and to lower cerebral infiltration of leukocytes in rodent models of ischemic stroke (Iadecola et al., 2001; Candelario-Jalil et al., 2007). COX-2-derived prostaglandin E2 may also contribute to ischemic cell damage by disrupting Ca^2+^ homeostasis in neurons through activation of EP1 receptors (Kawano et al., 2006). Interestingly, an association between functional outcome and specific COX-2 variants has recently been demonstrated in ischemic stroke patients (Maguire et al., 2011).

COX-2 has been implicated in hyperbaric oxygen preconditioning, since pharmacological inhibition of this enzyme abolishes the beneficial effects of the conditioning stimulus in a rat model of transient global cerebral ischemia (Cheng et al., 2011). Similarly, COX-2 induction participates to ischemic tolerance induced by cortical spreading depression (Horiguchi et al., 2006) or by a brief ischemic episode in rats (Choi et al., 2006; Pradillo et al., 2009), likely via the stimulation of the PGE2/PI3K/Akt pathway (Park et al., 2008). The elevated expression of COX-2 induced by ischemic preconditioning has been suggested to occur via a cascade involving epsilon protein kinase C and ERK1/2 activation, as well as NFkB nuclear translocation, as demonstrated *in vitro* (Kim et al., 2007, 2010).

While elevated expression of inducible NOS is associated with ischemic (Cho et al., 2005), TLR4-mediated (Pradillo et al., 2009) and anesthetic preconditioning (Kapinya et al., 2002), induction of this enzyme has also been implicated in the release of toxic amounts of NO by infiltrating neutrophils, microglia/macrophages and endothelial cells (Nakashima et al., 1995; Iadecola et al., 1996; Forster et al., 1999; Garcia-Bonilla et al., 2014b). NO released by endothelial cells early after the ischemic insult plays a beneficial role by inducing vasodilatation; whereas, at later stages, overactivation of neuronal NOS and, more importantly, *de novo* expression of inducible NOS contribute to ischemic tissue damage (Iadecola et al., 1996, 1997; Moro et al., 2004; Murphy and Gibson, 2007). In fact, excessive production of NO by inducible NOS is cytotoxic by promoting NFκB activation, by inhibiting ATP-producing enzymes, by producing peroxynitrite and by stimulating other pro-inflammatory enzymes such as COX-2 (Nogawa et al., 1998; Greco et al., 2011). Moreover, NO contributes to ischemic cell death via S-nitrosylation and, thereby, activation of glutamate receptor (GluR)-6 signaling (Yu et al., 2008) and MMP-9 (Gu et al., 2002).

NO plays a crucial role in cortical spreading depression-induced tolerance to transient focal cerebral ischemia in rats (Horiguchi et al., 2005). Moreover, recent findings have demonstrated the involvement of endothelial NOS in remote ischemic preconditioning (Peng et al., 2012), while neuronal NOS has been involved in the neuroprotection exerted by remote post-conditioning (Pignataro et al., 2013).

## Concluding remarks

Although ischemic stroke is a major cause of mortality and long-term disability worldwide (Go et al., 2014), current therapeutic approaches for its acute treatment only rely on blood flow restoration by thrombus lysis or removal (Mangiafico and Consoli, 2014; Berkhemer et al., 2015; Hacke, 2015). The therapeutic window, intended as the temporal range during which the endovascular treatment may reach the target of a useful recanalization, is conventionally set at a threshold of 6 h; therefore, only less that 10% of patients may actually benefit from these procedures. Thus, the identification of novel targets that allow widening the time-window for pharmacological intervention, as well as the possibility of limiting the development of ischemic brain damage by promoting innate beneficial responses, is a urgent challenge. To date, the clinical translation of immunomodulatory drugs has been hampered by the fact that most strategies tested in humans were purely based on anti-inflammatory approaches (Table 2), disregarding the beneficial roles of some elements of the immune reaction to stroke injury (Amantea et al., 2014a). In this context, targeting immune responses that evolve during hours or days after the ischemic insult, by selectively promoting their beneficial components, represents a promising avenue for the development of more effective and safe stroke therapeutics.

**Table 2:** **Acute ischemic stroke trials for the clinical validation of anti-inflammatory and immunomodulatory drugs**.

**Trial**	**Drug**	**Mechanism**	**Phase**	**Status**
Safety Study of Interferon Beta 1a for Acute Stroke	Recombinant human interferon beta-1a (IFN-β1a) (Rebif®)	Inhibition of pro-inflammatory cytokines production and prevention of blood brain barrier disruption	I	Completed
Intravenous immunoglobulin (IVIG) in acute ischemic stroke: a pilot study	Immunoglobulin	Scavenging active complement fragments	I	Withdrawn
Study of a neuroprotective drug to limit the extent of damage from an ischemic stroke (MINOS)	Minocycline	Anti-inflammatory/anti-apoptotic effects	I/II	Completed
Acute stroke therapy by inhibition of neutrophils (ASTIN)	Recombinant neutrophil inhibitory factor (UK-279, 276)	Blockade of neutrophil adhesion to endothelium	II	Terminated
E-selectin nasal spray to prevent stroke recurrence	E-selectin	Induction of mucosal tolerance to human E-selectin causing a shift of immune response from T(H)1 to T(H)2 type	II	Terminated
Study of interleukin-1 receptor antagonist in acute stroke patients	IL-1 receptor antagonist	IL-1β receptor blockade	II	Completed
Efficacy and safety of FTY720 for acute stroke	The sphingosine-1-phosphate receptor (S1PR) regulator Fingolimod (FTY720)	Reduced trafficking of T cells, B cells, NK cells, and other S1PR-expressing cells into the brain	II	Recruiting
Controlled study of ONO-2506 in patients with acute ischemic stroke	Arundic acid (ONO-2506)	Astrocyte modulating agent	II/III	Completed
Hu23F2G Phase 3 stroke trial (HALT)	Monoclonal antibody (humanized) against the neutrophil CD11/CD18 cell adhesion molecule, Hu23F2G (LeukArrest^®^)	Reduction of brain infiltration of neutrophils	Pilot III	Terminated
Enlimomab acute stroke trial (EAST)	Murine anti-ICAM-1	Blockade of leukocyte attachment and migration through cerebral endothelium	III	Completed
Neuroprotection with minocycline therapy for acute stroke recovery trial (NeuMAST)	Minocycline	Anti-inflammatory/anti-apoptotic effects	IV	Terminated

## Author contributions

All the authors participated in the collection, review, and analysis of the relevant literature, as well as to drafting and revising of the manuscript.

### Conflict of interest statement

The authors declare that the research was conducted in the absence of any commercial or financial relationships that could be construed as a potential conflict of interest.
